# Global Perspectives on the Management of Primary Progressive Aphasia

**DOI:** 10.21203/rs.3.rs-4100219/v1

**Published:** 2024-03-19

**Authors:** Jeanne Gallée, Jade Cartwright, Stephanie Grasso, Regina Jokel, Monica Lavoie, Ellen McGowan, Margaret Pozzebon, Bárbara Costa Beber, Guillaume Duboisdindien, Núria Montagut, Monica Norvik, Taiki Sugimoto, Rosemary Townsend, Nina Unger, Ingvild E. Winsnes, Anna Volkmer

**Affiliations:** Center for Psychometric Analyses of Aging and Neurodegeneration, Department of Medicine, University of Washington; School of Health Sciences, University of Tasmania; Department of Speech, Language, and Hearing Sciences, The University of Texas at Austin; Temerty Faculty of Medicine, University of Toronto; Chaire de recherche sur les aphasies primaires progressives – Fondation de la famille Lemaire, Université Laval; Pennine Care NHS Foundation Trust; Age Right Speech Pathology; Department of Speech, Language, and Hearing Sciences, Universidade Federal de Ciências da Saúde de Porto Alegre (UFCSPA); Chaire de recherche sur les aphasies primaires progressives – Fondation de la famille Lemaire, Université Laval; Alzheimer’s Disease and other Cognitive Disorders Unit, Neurology Service, Hospital Clinic Barcelona; Department of Linguistics and Scandinavian studies, University of Oslo; Center for Psychometric Analyses of Aging and Neurodegeneration, Department of Medicine, University of Washington; Dyscover Ltd.; Department of Neurology, University Medicine Greifswald; Department of Linguistics and Scandinavian studies, University of Oslo; Department of Psychology and Language Science, University College London

## Abstract

Speech-language therapists/pathologists (SLT/Ps) are key professionals in the management and treatment of primary progressive aphasia (PPA), however, there are gaps in education and training within the discipline, with implications for skills, confidence, and clinical decision-making. This survey aimed to explore the areas of need amongst SLT/Ps working with people living with PPA (PwPPA) internationally to upskill the current and future workforce working with progressive communication disorders. One hundred eighty-five SLT/Ps from 27 countries who work with PwPPA participated in an anonymous online survey about their educational and clinical experiences, clinical decision-making, and self-reported areas of need when working with this population. Best practice principles for SLT/Ps working with PwPPA were used to frame the latter two sections of this survey. Only 40.7% of respondents indicated that their university education prepared them for their current work with PwPPA. Competency areas of “Knowing people deeply,” “Practical issues,” “Connectedness,” and “Preventing disasters” were identified as the basic areas of priority and need. Respondents identified instructional online courses (92.5%), sample tools and activities for interventions (64.8%), and concrete training on providing care for advanced stages and end of life (58.3%) as central areas of need in their current work. This is the first international survey to comprehensively explore the perspectives of SLT/Ps working with PwPPA. Based on survey outcomes, there is a pressing need to enhance current educational and ongoing training opportunities to better promote the well-being of PwPPA and their families, and to ensure appropriate preparation of the current and future SLT/P workforce.

## 1 Background

Worldwide, it is estimated that a person receives a diagnosis of dementia every three seconds, and 78 million people are projected to be affected by dementia by 2030^1^. Despite these staggering figures, current infrastructures to provide proactive and longitudinal medical care to this population are frequently insufficient. Between 50–80% of dementia cases go undocumented in primary care in high income countries^2^, a statistic that only rises in countries classified as low to middle income^1–3^. In the words of the World Health Organization (WHO) Director General, dementia is a “looming… public health disaster, ‘a tidal wave’”^3,4^.

Dementia is classified as young onset when it occurs before the age of 65^5–7^. A recent systematic review by Hendriks et al. (2023) determined that approximately 370,000 people worldwide are diagnosed with young-onset dementia per year. While a much smaller percentage, the prevalence of young-onset dementia and far-reaching societal implications are significant. Young-onset dementias, ranging from Alzheimer’s disease to frontotemporal dementia to Parkinson’s disease, insidiously disrupt mechanisms of communicative competence^8–19^ and therefore warrant speech and language intervention. Primary progressive aphasia (PPA) is a distinctive subtype of young-onset dementia syndromes that selectively impacts functions of speech and language at the milder stages of the condition^20–22^. Identifying the onset of PPA, or its milder stages, poses significant challenges due to the relative complexity and rarity of the condition^20–22^. In the absence of a one-to-one correspondence between presentation and underlying pathology, PPA can present as one of three established variants (semantic, nonfluent, and logopenic) or heterogeneously, where symptomatology and evolution of presentation can vary starkly^20–22^. Despite these differences, the functional impact of PPA encompasses all variants: a fundamental change and progressive loss to communicative ability.

The capacity to communicate is an imperative feature of the human experience. A behavioral approach to the care of people living with PPA (PwPPA) is paramount to enhance, maintain, or compensate for communication loss. Presently, there is no curative treatment publicly available for this condition^22^. Speech and language therapy is the primary intervention that can slow down the behavioral impact of symptoms, which immediately impacts positive engagement, participation, and quality of life. Such intervention has been shown to maintain communication abilities for longer through both compensatory and restitutive approaches^23–25^. Early referral to speech and language therapy is imperative to optimize possible improvements, maintenance, and compensation for decline in linguistic capacity faced by PwPPA^26–29^. Speech-language therapists/pathologists (SLT/Ps) are recognized as central specialists in the care of PwPPA with roles in the diagnosis and coordinated care of the condition^22,27,28, 30, 31^. Kate Swaffer, founder of Dementia Alliance International (DAI), and person living with the semantic variant of PPA, has spoken publicly about the pressing need for SLT/Ps to be consistently integrated in the care of those living with the condition.

“People with dementia who will almost all have language and speech changes, need proactive speech pathology after diagnosis, not just a speech pathologist to attend when we can no longer swallow well nearer to the end of life… this is how I have managed to still speak as well as I do.”^32^.

Despite this need, there remains a consequential gap in the number of people living with dementia who qualify for speech and language therapy and those that receive this service^27,33,34^. Evidence demonstrates that PwPPA must begin speech and language intervention as early as possible to avoid the risk of not benefitting from impairment-based or functional interventions^26,33^. Furthermore, as highlighted by Swaffer (2019), people living with early-onset dementias, such as PPA, and their families seek support to optimize participation and quality of life outcomes, which is bolstered by appropriate and timely access to SLT/P intervention^31,35^. Perhaps more critically, beyond gaps in referrals, SLT/Ps report limited confidence and feel underprepared for working with PwPPA^34,36,37^.

In response to these concerns, a group of international expert clinician-academic SLT/Ps worked collaboratively to establish best-practice principles to guide SLT/P services for PwPPA and their families^38^. The consensus process culminated in a set of seven best practice principles visualized in the form of the “Clock Model”. With the aim of promoting autonomy and person-centered care, these principles represent concepts of “Knowing people deeply,” “Preventing disasters,” “Practical issues,” “Professional development,” “Connectedness,” “Barriers and limitations,” and “Peer support and mentoring towards a shared understanding” (see Table 1). The principles provide a framework for the essential components of SLT/P intervention and can be used to guide clinical decision-making, care coordination and service development in the SLT/P field. Further, use of the principles can be extended to the professional development, training and education needs of the current and future SLT/P workforce. Utilizing the principles of the aforementioned aspects of SLT/P practice has the potential to close the current gap between the expressed need for SLT/P services by PwPPA and their families, and the readiness of SLT/Ps to provide best-practice care and innovation in the PPA field.

Furthermore, the best practice principles can guide the development of educational modules and training tools for future and current SLT/Ps seeking to provide consistent and evidence-based care for PwPPA. They can also serve as a starting point for maximizing the care for communication loss available to the broader spectrum of dementias. There are significant translational applications of this work on PPA as communication challenges are experienced across neurodegenerative conditions^39,40^ and people with non-language led dementias and their families identify SLT/Ps as essential in providing them with the relevant support^41^. While PPA is currently the only recognized language-led dementia syndrome, enhancing current SLT/P education, training, and services will allow us to upskill capacity within the profession and generalize these skills to a broader population. This is especially relevant as the functional impact of language impairment in dementia has yet to be fully characterized^40^: while decline in linguistic function in progressive conditions is well-documented^42,43^, the clinical relevance of these changes, and how these can be addressed through SLT/P intervention, remains to be widely recognized.

To develop a relevant and viable resource, gaps in current educational opportunities and exposure to aspects of the PwPPA care continuum must be addressed. As such, the aim of this study was to investigate a more global perspective on SLT/P confidence in enacting the roles and responsibilities within each of these principles, as well as clinical prioritization and ranking of basic competencies when working with PwPPA. To accomplish this, we conducted a survey to explore the experiences of SLT/Ps working with PwPPA with reference to their clinical settings, educational experiences, clinical decision-making, and self-reported areas of need in current practices of care.

## 2 Methods

### 2.1 Survey Structure

The survey is reported in line with the CHERRIES guidelines for electronic surveys^44^. Survey items were developed by a core set of the study authors: JG, JC, RJ, ML, EM, MP, and AV (Appendix A). Data was collected anonymously, where IP addresses were suppressed, via the Qualtrics^®^ (Qualtrics, Provo, UT) survey platform and respondents had the option to review their responses and skip over any survey items. The survey structure consisted of five parts: (1) identity, (2) clinical setting and exposure to PPA, (3) educational experiences, (4) clinical decision-making, and (5) areas of need. The first sections were predominantly open-ended for respondents to fill out. For clinical decision-making, survey items were created to investigate clinician confidence, prioritization, and ratings of basic competency for each of Volkmer, Cartwright, Ruggero et al.’s (2023) best practice principles. Confidence was ranked on a five-point scale (0 = not at all confident, 1 = slightly confident, 2 = somewhat confident, 3 = confident, 4 = entirely confident). Priorities and basic competencies were ranked on a 7-point scale (1 = highest, 7 = lowest). Areas of need included multiple choice questions related to the areas of educational needs and the adaptation of existing tools for PPA, followed by the open-response question, “What are three things you wish you knew when you first started working with PPA?”. Survey items were translated by native speakers of French (ML and GD), German (NU and JG), Japanese (TS), Norwegian (MN and IEW), Portuguese (BCB), and Spanish (SG).

### 2.2 Survey Distribution

Ethical clearance for this work (project ID: STUDY00017617, PI: Jeanne Gallée) was obtained on March 23rd, 2023 from the University of Washington Human Subjects Division (HSD). All methods were performed according to relevant guidelines and regulations. Informed consent was obtained from each study participant. Data collection was conducted by method of network sampling between April 13th, 2023 and January 31st, 2024. SLT/Ps were recruited using a snowball method. Announcements for the study were disseminated on social media platforms (including but not limited to X and relevant speech-language communities on Facebook), American Speech-Language-Hearing Association (ASHA) Sig 2 online discussion boards, and emails to viable contacts within user networks. Initial distribution of the survey occurred through direct contact with the International SLT/P PPA Network^38,45^, where working members shared the survey with their own professional networks in African, European, and North American countries. Professional associations that represented speech-language pathologists around the world were contacted by the first author. These included the ‘Adult and elderly Language Working Group’ of the Brazilian Speech-Language Pathology and Audiology Society, Colegio de Fonoaudiología de Chile, Colombian Association of Phonoaudiology, Deutscher Bundesverband für Logopädie e.V., Deutschschweizer Logopädinnen- und Logopädenverband, Emirates Speech-Language Pathology Society, Hong Kong Association of Speech Therapists, Indian Speech and Hearing Association, Israeli Speech, Hearing and Language Association, Österreichische Gesellschaft für Logopädie, Phoniatrie und Pädaudiologie, Russian Public Academy of Voice, Saudi Society of Speech-Language Pathology and Audiology, Speech Pathologists and Audiologist Association in Nigeria (SPAAN), Speech Pathology Australia, Speech-Language and Audiology Association of Trinidad and Tobago (SLAATT), South African Speech Language Hearing Association (SASLHA), and Thai Speech-Language and Hearing Association. Additional centers for speech and language services in Germany, Japan and Kenya were contacted.

### 2.3 Survey Analysis

A mixed-methods approach was implemented to analyze the quantitative and open-ended feedback. Descriptive statistics were used to aggregate quantitative survey responses. Open-ended survey responses were initially translated to English via Google Translate and then validated or hand checked and corrected by the writers of the original survey. Three coders (JG, JC, and AV) performed content analyses to identify patterns in reported SLT/P experiences and needs. The best practice principles were used as a predefined set of categories to code for content. Consensus was established on 10% of open-ended responses following independent coding. The remainder of the response codes were then collated by the first author (JG).

## 3 Results

Three hundred thirty-seven users clicked upon the survey link and 188 provided consent. Where respondents failed to provide consent all consequent responses to any survey items were excluded from the analysis. An additional three respondents who reported to not be SLT/Ps were excluded. Data was collected from 185 respondents across 27 countries (see Table 2) in seven languages and included in the analysis. Of the 185 participants included in the analysis, the average survey completion rate was 84.2% (SD: 25.1%), where 97 users completed the survey in English, 29 in French, three in German, 13 in Japanese, 17 in Norwegian, 10 in Portuguese, and 17 in Spanish.

### 3.1 Identity

**Figure 1:**
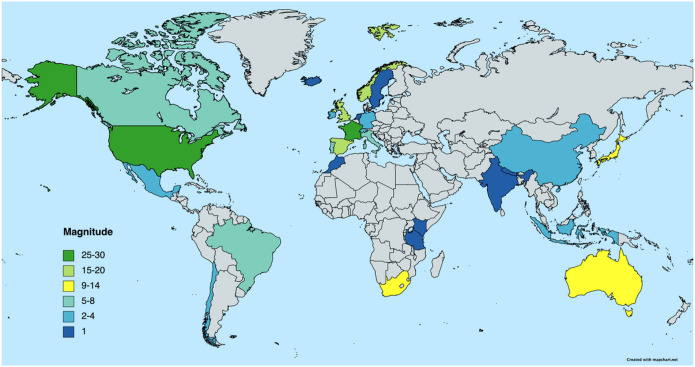
Global distribution of participant sample. Results consisted of 185 respondents from 27 countries from six continents. Map was created through mapchart.net.

Ninety-eight users reported to be based in Europe, 36 in Northern Anglo-America, 19 in Asia, 13 in Africa, 11 in Latin America, and 9 in Oceania (see Fig. 1 and Table 2). One hundred sixty-two participants described themselves as female, 21 as male, and 1 as non-binary. The average age was 38.2 years (SD: 11.2). Eighty-eight respondents identified as monolingual, where the average number of languages spoken was 1.87 (SD: 1.08). Finally, 124 respondents described themselves as monocultural.

### 3.2 Clinical Practice

Fifty-eight percent of respondents reported to work in an urban setting that was either private practice (45.0%) or a hospital setting (38.3%). In these clinical settings, 22.3% of respondents reported to work in more than one language. Most respondents (62.4%) reported to work in an interdisciplinary team, where 36.5% worked in a team of SLT/Ps and 23.5% as independent/sole charge clinicians (17.8% of respondents reported to work in various configurations). Respondents reported to have worked an average of 11.8 years (SD: 9.56, range: 0–43.0) as SLT/Ps, with 6.00 years (SD: 6.29, range: 0–30.0) of exposure to PwPPA. In their work setting, 81.8% reported to have had a SLT/P mentor, where 31.1% affirmed that this mentor had experience with PwPPA. Respondents reported to know approximately seven other SLT/Ps working with PPA (SD: 7.98), across their local and global networks of colleagues.

### 3.3 Education

Twenty-four percent of respondents stated that they first learned about PPA through their place of work, 59.2% during their university education (either Bachelor’s or Master’s), 6.51% in a clinical placement, and 5.91% during their doctoral training. Eighteen percent reported that their highest degree was a bachelor’s or vocational equivalent, 53.3% a master’s, and 18.6% a doctoral degree. Most respondents estimated to have received less than two hours of education on PPA during their academic training (52.1%), where only 10.1% affirmed to have received more than five hours. Thematically, 42.9% stated that these educational hours covered processes of assessment of PwPPA, 30.4% interventions for PPA, and 12.5% counseling specific to the needs of PwPPA (with 32.7% reporting to have learned about counseling more generally). Of these respondents, only 40.7% affirmed that their university-level education was beneficial to their current work with PwPPA and 28.0% to have had a clinical placement with PwPPA in the context of their SLT/P training. Finally, 50.0% disclosed having sought out professional development opportunities specific to PPA and 48.5% shared that they were members of a PPA study group. Global distributions of the topics covered in university-level education for speech and language therapy can be seen in Table 3. Global regions were defined as the divisions proposed by the United Nations^46^: Africa, Asia, Europe, Latin America and the Caribbean, Northern America, and Oceania.

### 3.4 Clinical Decision-Making

**Figure 2:**
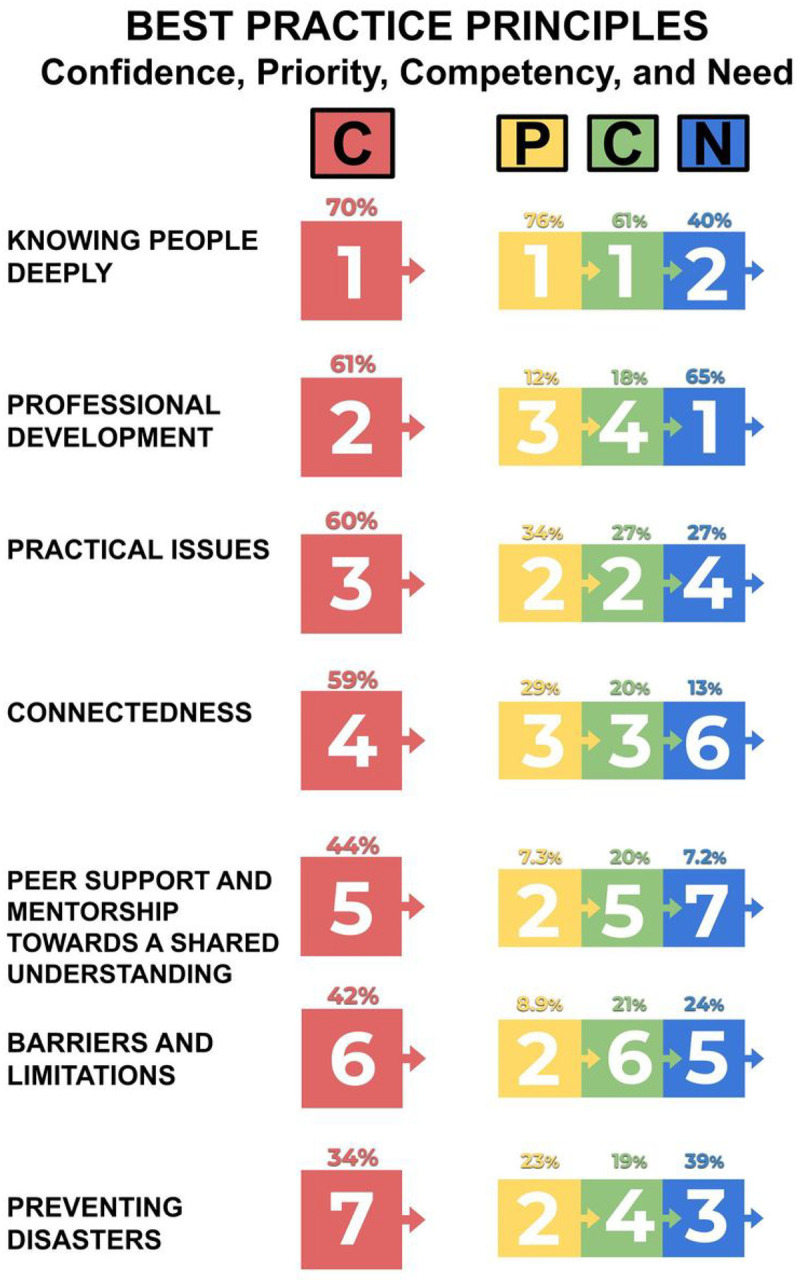
Rankings of confidence, priority, competency, and need for the seven best practice principles (1 = highest and 7 = lowest). Percentages indicate the agreement of participants for each numerical rank. Illustrated in the far-left column (red), respondents ranked themselves to be most confident in “Knowing People Deeply” and least confident in “Preventing Disasters”. In the second column (yellow), the most agreement was found for “Knowing People Deeply,” “Practical Issues,” and “Connectedness” as top priorities for a SLT/P working with a new client with PPA, with minimal agreement on priorities for the remaining principles. Basic competency rankings of the best practice principles, indicated in the third column (green), revealed strongest support for “Knowing People Deeply,” “Practical Issues,” and “Connectedness” as top competencies for SLT/Ps working with PwPPA. Finally, thematic analyses of areas of need in the far-right column (blue), revealed that “Professional Development,” “Knowing People Deeply,” and “Preventing Disasters” were ranked as in most critical need of additional support and resources for SLT/Ps.

**Figure 3:**
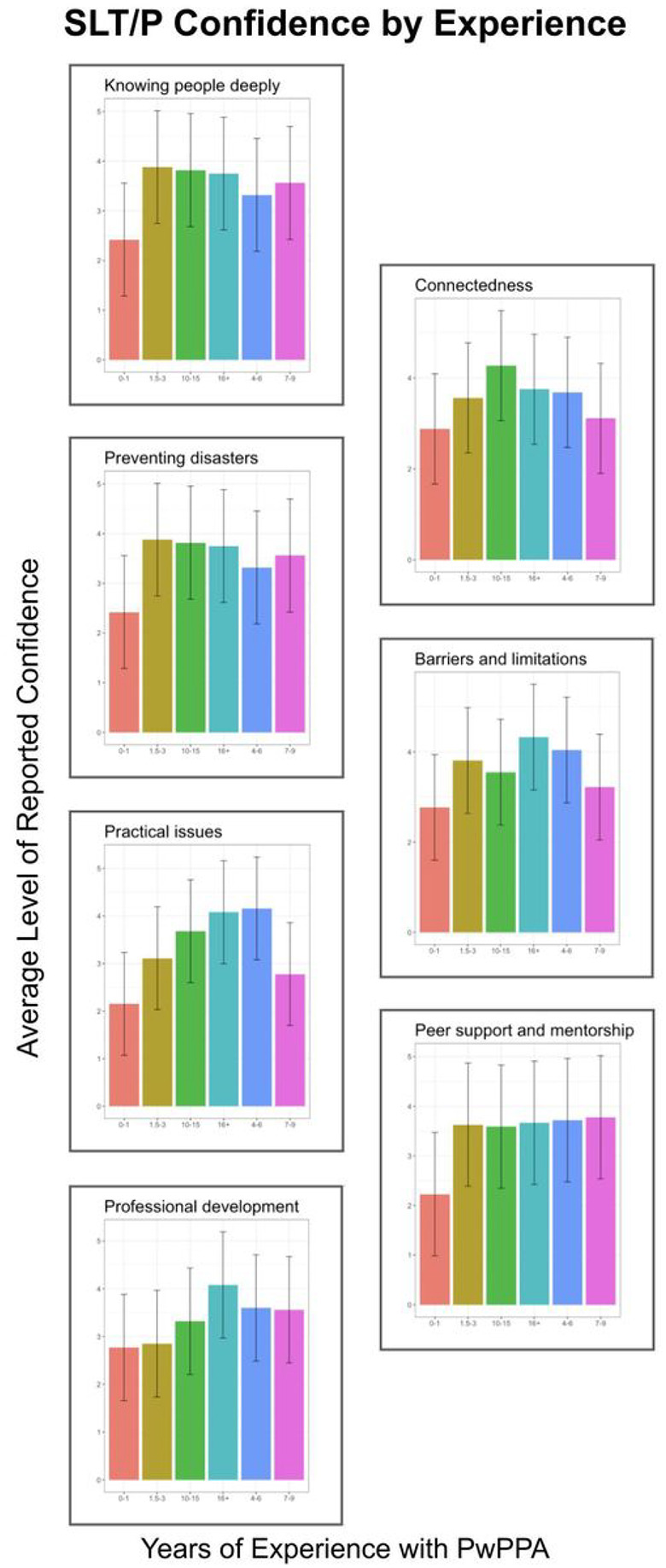
Reported SLT/P Confidence by Experience. For each best practice principle, SLT/P confidence is represented by the relative years of experience of working with PwPPA. Average reported levels of confidence ranged between slightly confident and confident, with average upwards shifts towards somewhat confident seen by the time 4 to 6 years of experience were gathered.

The majority of respondents reported being confident to entirely confident on the best practice principles of “Knowing people deeply” (69.9%), seeking “Professional development” opportunities (60.9%), and “Practical issues” (60.2%; see Fig. 2). In contrast, the majority of respondents felt somewhat or less confident in “Preventing Disasters” (65.0%), “Barriers and limitations” (56.9%), and “Peer support and mentorship towards a shared understanding” (51.5%). Confidence levels varied by years of clinical experience working with PwPPA (see Fig. 3). At career onset (0–1 years), confidence averaged at slightly to somewhat confident for all best practice principles (average: 2.59, SD: 0.31, where 0 = not confident, 3 = somewhat confident, 5 = extremely confident). When comparing reported confidence at career onset with 4 to 6 years of experience, the average upwards shift in confidence was 1.15 (SD: 0.26, range: 0.84–1.53), representing a shift from slightly/somewhat confident to confident for “Knowing people deeply” and “Connectedness,” slightly/somewhat confident to somewhat confident for “Preventing disasters,” “Practical issues,” “Professional development,” “Barriers and limitations,” and “Peer support and mentorship towards shared understanding.” In comparison, the average shift from career onset to 10 to 15 years of career experience was 1.10 (SD: 0.24, range: 0.82–1.44), representing the same patterns per principle as the comparison to 4 to 6 years. Principle confidence by regional location of respondents can be seen in Table 4.

For clinical prioritization for a new client with PPA, the majority ranked “Knowing People Deeply” as the top priority (76.4%), “Practical Issues” as the second (34.1%) and “Connectedness” as the third (29.3%). Finally, respondents ranked these same principles as the top three basic competency areas for SLT/Ps working with PwPPA, where “Knowing People Deeply” received 60.9% support, “Practical Issues” 26.8%, and “Connectedness” 20.3%.

### 3.5 Areas of Need

Where respondents were asked to indicate the areas of their practice in which they may need more knowledge, access to resources, or support in their work with PwPPA, the top two reported areas of need were designing intervention approaches for PwPPA (59.3%) and providing care for advanced stages and end of life (58.3%; see Table 5). Furthermore, when asked to choose from a selection of tools that they deemed helpful and used in other areas of practice that they wished to have for their work with PwPPA, respondents selected instructional online video courses (92.5%), sample tools and activities for interventions (64.8%), and sample goal banks delineated by PPA variant (57.5%).

All open-ended responses to the question “What are three things you wish you knew before working with PPA” aligned with one of more of the seven best practice principles, characterizing respondent reported educational and professional development needs by principle. The three most frequent themes of the open-response coding were “Professional development” (65.1%), “Knowing people deeply” (39.8%), and “Preventing disasters” (38.6%). These were closely followed by “Practical issues” (26.5%) and “Barriers and limitations” (24.1%). Finally, “Connectedness” (13.3%) and “Peer support and mentorship towards a shared understanding” (7.23%) were coded least frequently in the qualitative responses.

#### 3.5.1 Coding of Open-Ended Responses

##### 3.5.1.1 Professional development

Foundational knowledge about the diagnostic process of PPA, the three variants, and how these fit into the schema of neurodegenerative conditions was highly sought after. Such knowledge is imperative to meet most principles, from “Knowing people deeply” to “Practical issues” as it relates to tailoring intervention plans for the now and future. Basic educational gaps were highlighted in respondents’ concerns in relation to “what do you wish you’d known?:

About the condition. The trajectory and natural history of the PPA conditions.

I wish I had more basic medical knowledge about the disease because I feel it helps a lot with understanding the symptoms and their evolution, not restricted to language

“That the diagnostic categories and labels were only provisional. I have seen “atypical dementias” with communication disorders shift their variants not only as the patients progressed but also as the criteria for classification changed. E.g., “logopenic.” A little humility would have been welcome - even the acknowledgement that while stroke, tumor and traumatic types of damage resulting in aphasia have had centuries of examination, the progressive neuronal diseases resulting in aphasia were in the infancy of their aphasiology”

##### 3.5.1.2 Knowing people deeply

Implementing a person-centered approach to speech and language services was a fundamental feature of the responses. Respondents particularly identified professional development needs related to establishing a relationship with a patient and their support systems, as well as how to adapt therapeutic intervention on an individual basis:

It’s about the person, and not only the disease.

Adjusting to client and support network preferences, as well as the needs of the condition, was also underscored.

Support is an ongoing and evolving journey. It can take many forms and is shaped by the client and their resources (geographic, financial, familial support, and personal factors that shape their requests, needs, and preferences).

Promoting the autonomy of PwPPA was also felt to be a critical skill:

Skills to provide relational care. Coaching skills and how to empower self-management. How to provide education and information in a tailored and enabling way.

##### 3.5.1.3 Preventing disasters

The open-ended responses emphasized the complex concerns and needs that must be addressed when building workforce capacity. Many respondents shared apprehension and felt ill-equipped to provide anticipatory care or to respond to the emotional and ethical issues that arise when working with a progressive condition.

“I would have been more upfront about the area of “preventing disasters” - I was a new clinician at the time and not yet entirely comfortable with broaching subjects related to death and decline. It would have been key to get these conversations and decisions going ASAP before further language decline occurred, making the conversations more difficult.”

Respondents identified specific areas of need as:

How to provide counselling related to the progressive nature of the disease.

“People have the right to dignity of risk.”

How to provide therapy that focuses on prevention.

##### 3.5.1.4 Practical issues

Practical concerns, such as the kinds of goals to target in speech and language therapy, and when to introduce compensatory or functional goals, were among the top concerns voiced by respondents:

Functional goals trump linguistic ones! These should be introduced in parallel to the possibility of restorative intervention goals and early on, in fact, immediately.

These responses paralleled the reported need for concrete guidance in the form of sample goals, interventions, activities; similarly, responses mapped on the reported need for professional development modules on informational counseling and education for both patients and families:

How to provide AAC intervention in a preventative/preparatory manner.

Functional goals may be very simple but complex to support patients/families.

##### 3.5.1.5 Barriers and limitations

The challenge of receiving a diagnosis, and the associated delays, was also mentioned. Responses such as these highlight the necessity for early and accurate diagnosis of the condition, which is dependent on immediate referral to neurology and SLT/P services and the expertise of the SLT/P in identifying progressive language change.

It is difficult to help … when they are diagnosed late.

Participants highlighted that the SLT/P is often the provider that connects PwPPA and their support partners to other services or community supports. Furthermore, respondents shared the relative burden of often being the provider with the most knowledge of the functional impact of PPA, whilst also needing to define and advocate for the SLT/P role to other healthcare providers.

That I as the SLP may be the greatest advocate for my patients and that I may be the expert on the impact of their condition.

The relative difficulty of explaining a diagnosis, or describing the speech and language concerns of, within the SLT/Ps scope without the support of other providers, was also described.

How to give a diagnosis when you are the only likely medical professional who knows about the problem.

##### 3.5.1.6 Connectedness

Apprehension and inexperience in identifying, establishing, and maintaining connection between a patient and their community and amongst the interdisciplinary healthcare team were common themes relating to responses that fell under the “Connectedness” principle coding.

I have to work to convince neurologists and other healthcare providers of my role in helping PwPPA.

The necessity to connect with the interprofessional team of each client to ensure continuity of care, eliminate miscommunication and delays in service delivery, and enhance shared understanding of the client and their care needs.

##### 3.5.1.7 Peer support and mentorship towards a shared understanding

Finally, respondents shared concerns about apparent knowledge gaps about the functional impact of PPA and how this posed challenges their role as SLT/Ps in the context of the multidisciplinary team.

That the neurologists and neuropsychologists were—as a generalization—over-confident in their state of practice, and that SLPs were not using our voice, nor being given the credibility we had earned.

Frustration with the perceived lack of support, especially as it pertains to counseling, was reported:

These people present with great psychological suffering and I often feel helpless. The psychologists around me do not take care of them because of their language disorders, even if an AAC tool is put in place.

The apparent lack of university-level and ongoing educational resources for SLT/Ps working with PwPPA was also emphasized:

Where to find a comprehensive set of helpful resources, such as case examples and approaches?

## 4 Discussion

This study characterizes the international perspectives of the SLT/P workforce and examines its professional development needs in relation to working with PwPPA and their families. The survey outcomes reveal compelling evidence that current educational, training, and ongoing support opportunities for SLT/Ps to develop their knowledge and skills in working with PwPPA, a clinical syndrome of dementia, necessitate refinement. Our findings demonstrate a shortfall in the preparedness and professional support capacity that SLT/Ps receive internationally, characterized by insufficient time spent on PPA in university courses, infrequent or rare exposure to PPA during clinical placements, limited access to mentors that are experts in the condition, and an expressed need for more functional and specialized resources for the condition. These concerns justify a global need for a more adequately prepared and supported SLT/P workforce to ensure best practice pre and post-diagnostic care for PwPPA and their families. The findings support use of the best practice principles^38^ to guide the systematic development of education, training and professional support opportunities for the SLT/P profession. Investing in the SLT/P workforce is timely and significant. With the current and expected global “tidal wave” of people living with dementia^4^, an upscaled framework of support is critical to ensure the needs of PwPPA and their families can be met.

Our outcomes represent a global perspective on needs worldwide. Seven of the 27 countries represented in the survey outcomes were low to middle income^47^, globally distributed in Africa, Asia, and Latin America and the Caribbean. Differences in educational experiences and reported confidence in the best practice principles emerged by region of practice and experience. Respondents from Asia reported the lowest levels of confidence per principle, whereas those from Latin America and the Caribbean, Northern America, and Oceania consistently reported the highest levels. Confidence levels in Europe fell closely behind these, with respondents from Africa reporting the second-lowest levels. While the relative overrepresentation of European countries must be taken in consideration here, higher levels of usefulness of education were reported by respondents from Europe and North America. Across all participants, confidence increased with experience, peaking after a few years of practice with PwPPA.

This study extends the consensus work on best practice principles^38^ by revealing a global perspective on key competency issues: including “Knowing people deeply,” responding to “Practical issues,” promoting “Connectedness”, and “Preventing disasters”. Respondents expressed the need for practical guidance and training to diagnose and understand the natural history of PPA, and manage the more complex, evolving, ethical, and psychosocial aspects of PPA, for example, supporting care partners, promoting autonomy, and helping PwPPA and their families plan for the future. While no respondent expressed doubt about the importance of speech and language intervention for PwPPA, over half indicated that designing intervention approaches was a primary gap in knowledge and requested sample goal bank materials as well as exemplars of tools and activities to approach these, delineated by PPA variant. This was a common recommendation in the open-ended responses, with SLT/Ps advocating for direct instruction in the range of evidence-based intervention options for PwPPA (including both impairment-focused and more functional, compensatory interventions), as well as direct skill-building opportunities to deliver interventions, such as AAC, communication partner training and counselling. Respondents ranked “Preventing disasters” as a top priority and area of need while reporting the lowest levels of confidence in this aspect of care. As proactive and anticipatory care is essential for PwPPA to maximize quality of life outcomes across the continuum of care, clear clinical guidance to meet this need is required. With appropriate preparation, SLT/Ps can build capacity within the healthcare systems in which they operate, allowing them to then build capacity and support other members of each patient’s healthcare team.

## 5 Future directions

The findings of this research highlight the pressing need to develop education, training and professional development opportunities for SLT/Ps working with people with PPA around the world to facilitate best practice care and to optimize quality of life outcomes. Further, the findings support use of the Clock Model and best-practice principles to inform this work, developing training modules and skill-building experiences that support competency development in “Knowing people deeply”, Preventing disasters”, “Connectedness” and “Practical Issues”. This would identify theories, intervention approaches, tools and clinical skills that align with each principle and that promote proactive and responsive person-centered care. With the work described here, the International SLT/P PPA Network has taken concrete steps to identify gaps in educational and professional development opportunities to upscale the current and future SLT/P workforce and enhance the care provided to PwPPA. Our goal is to also build public awareness of PPA and the SLT/Ps role in treating this condition to boost the overall care received by this population.

The outcomes of this work also highlight the ongoing barriers PwPPA face when accessing SLT/Ps and those that SLT/Ps face within their profession in providing optimal patient care. As a result, further work to probe the source of referral bottlenecks and limited understanding of the SLT/Ps role in the care of PwPPA is warranted. As an immediate next step, we recommend surveys of general practitioners, neurologists, and geriatricians who work with PwPPA. Through this research and the collaborative efforts of our international research team and the broader international PPA network we are well positioned to respond to the identified workforce needs and build current and future workforce capacity. Working groups will be established to provide professional development resources and opportunities. Further, our newly established website will provide a critical forum for sharing resources and information to support best-practice care. We recommend that, as a future direction, professional SLT/P associations define and disseminate competencies for SLT/Ps, as supported by the educating bodies, to work with this population. Finally, survey respondents indicated various gatekeepers and the need to explore barriers in accessing SLT/P services. Further characterization of the perspectives of general practitioners, neurologists, neuropsychologists, and other vital team members in the care of PwPPA, is needed to identify and hopefully reduce possible obstacles in the comprehensive care necessitated by this condition.

These collective efforts will have obvious benefits for PwPPA and their families, as well as the wider community of people living with progressive aphasia and other types of dementia. Our work will be incomplete without the inclusion of PwPPA and their families, thus motivating our future stakeholder collaborations to improve our resources.

## Figures and Tables

**Table 1:** A description of the best practice principles for speech-language therapists/pathologists working with primary progressive aphasia (Volkmer, Cartwright, Ruggero et al., 2023).

Principle	Applications
Knowing people deeply	Understanding a person’s values, environment, and systems of support to explain a diagnosis, tailor collaborative and person-centered plans of care, and provide informational counseling
Preventing disasters	Providing timely specialist referrals and anticipatory care
Practical issues	Working with person and the people who surround them to identify possible barriers to enhance long-term management and deliver accessible and comprehensive care
Professional development	Refining personal understanding, skills, practices, and role in service provision
Connectedness	Connecting to other service providers or peer groups to optimize interdisciplinary support
Barriers and limitations	Identifying and advocating to address gaps, limitations, and failures in diagnoses, management, referrals, and interdisciplinary care
Peer support and mentorship towards a shared understanding	Providing training and peer mentorship to ensure ongoing education and improvement of practice

**Table 2:** Self-reported information on identity and clinical practice. Respondents could report multiple team compositions, locations, and settings for their clinical practice.

Total Respondents		185
**Age (SD, Range)**		38.2 (11.2, 23.0–85.0)
**Gender Identity**	Female	88.0%
	Male	11.4%
	Non-binary	.543%
**Cultural Identity**	Monocultural	66.8%
	Bicultural	24.5%
	Unspecified	8.70%
**Clinical Practice**		
**Years Practiced (SD, Range)**	General Experience	11.8 (9.56, 0–43.0)
	PPA Experience	6.00 (6.29, 0–30.0)
**Estimated % of PwPPA on Monthly Caseload (SD, Range)**		16.3% (25.4%, 0–100%)
**Team Composition**	Independent/Sole Charge Clinician	23.9%
	Team of SLT/Ps	32.8%
	Interdisciplinary Team	63.9%
**Location**	Urban	58.3%
	Rural	13.9%
	Mixed/Both	30.6%
**Setting**	Acute Care	10.0%
	Charity/Volunteer	7.22%
	Hospital	38.3%
	Inpatient Unit	17.8%
	Outpatient Rehabilitation	27.8%
	Private Practice	45.0%
	Research Facility	18.3%
	Residential Aged-Care Facility	11.1 %
	University Clinic	18.9%
	Other	14.4%
**Region of Practice**		**n**
Africa		13 (7.03%)
	Kenya (1), Morocco (1), Tanzania (1), South Africa (10)	
Americas		53 (28.6%)
	Brazil (7), Canada (6), Chile (2), Mexico (2), United States of America (36)	
Asia		19 (10.3%)
	Hong Kong (2), India (1), Indonesia (2), Japan (13), Nepal (1)	
Europe		98 (52.9%)
	France (25), Germany (3), Iceland (1), Ireland (2), Italy (8), Netherlands (1), Norway (17), Portugal (6), Spain (15), Sweden (1), Switzerland (2), United Kingdom (16)	
Oceania		9 (4.86%)
	Australia (9)	

**Table 3:** Self-reported topics of university-level education in speech and language therapy specialized to PPA by global region.

Region	Assessment	Intervention	Counseling	Education Reported to be Beneficial for Current Work with PwPPA
Africa	35.7%	21.4%	7.14%	21.4%
Asia	52.6%	42.1%	26.3%	36.8%
Europe	38.1%	27.8%	8.25%	35.1%
Latin America and the Caribbean	7.14%	7.14%3	0.00%	21.4%
North America	55.6%	36.1%	19.4%	33.3%
Oceania	0.00%	0.00%	0.00%	0.00%
Across All	42.9%	30.4%	12.5%	40.7%

**Table 4:** Reported confidence in best practice principles by global region. Acronyms represent the seven best practice principles (KPD = Knowing people deeply, PrD = Preventing disasters, PI = Practical issues, PD = Professional development, Co = Connectedness, BL = Barriers and Limitations, and PS = Peer support and mentorship towards a shared understanding). Confidence could range from 1–5, where 1 = “not at all confident” and 5 = “entirely confident”.

Region	KPD	PrD	PI	PD	Co	BL	PS
Africa	3.43 (0.79)	3.29 (0.95)	3.57 (0.98)	3.14 (0.90)	3.29 (0.76)	3.00 (115)	2.86 (0.90)
Asia	2.31 (135)	2.00 (1.26)	2.19 (1.22)	2.25 (1.24)	2.38 (141)	2.13 (1.26)	2.00 (1.10)
Europe	3.93 (0.77)	2.98 (1.08)	3.55 (1.00)	3.69 (0.94)	3.69 (115)	3.20 (1.10)	3.33 (1.19)
Latin America and the Caribbean	4.67 (0.52)	4.00 (0.89)	4.00 (0.63)	4.33 (0.82)	3.83 (117)	3.83 (0.75)	4.33 (0.82)
Northern America	4.19 (0.79)	3.16 (0.93)	3.87 (0.81)	4.00 (0.93)	3.81 (105)	3.13 (1.12)	3.29 (1.10)
Oceania	4.29 (1.11)	4.00 (0.58)	4.00 (0.58)	4.14 (0.69)	3.71 (0.95)	3.86 (0.90)	4.00 (0.82)
Across All	3.81 (107)	3.02 (1.13)	3.50 (1.08)	3.61 (1.11)	3.53 (1.21)	3.09 (117)	3.20 (1.24)

**Table 5:** Self-reported areas of need for SLT/Ps working with PwPPA and the tools SLT/Ps request based on those that exist for other populations.

Rank	Gap in knowledge, access, or support	%
1	Designing intervention approaches for PwPPA	59.7%
2	Providing care for advanced stages and end of life	55.5%
3	Providing family and care partner education	54.6%
4	Providing intervention/assistance with technologies and AAC devices	52.9%
5	Providing counseling to family and care partners of PwPPA	46.2%
6	Understanding and/or identifying a diagnosis	44.5%
7	Providing counseling to PwPPA	41.2%
7	Finding continuing education opportunities for working with PPA	41.2%
7	Advocating for reimbursement, coverage, or funding for PwPPA receiving SLT/P services	41.2%
10	Establishing goals of intervention for PwPPA	40.3%
11	Providing client education	39.5%
12	Providing education or advocating for your unique role as a SLT/P in the care of PwPPA	32.8%
13	Interacting with other healthcare professionals involved in the care of PwPPA	29.4%
14	Referring to other healthcare professionals	15.1%
Rank	Requested tools that exist for other populations	%
1	Online video courses (e.g., via MedBridge [U.S.A.-based continued education resources], internet-based instructional videos for different communication disorders)	84.0%
2	Sample tools and activities for intervention	63.0%
3	Sample goal banks for each PPA variant	54.6%
4	Access to a SLT/P support network	51.3%
5	Printable brochures related to diagnostics and information for families	47.1%
6	In-person or online workshops related to the diagnosis, assessment, and SLT/P intervention for PPA	45.4%
7	Access to a PPA support network geared towards clients	44.5%
8	Lived experiences of PwPPA (e.g., via documentary, biographical videos or short articles)	40.3%
Rank	Gap in knowledge, access, or support	%
9	Journal articles	26.9%
10	Textbooks	22.7%
